# Models of Experimentally Derived Competitive Effects Predict Biogeographical Differences in the Abundance of Invasive and Native Plant Species

**DOI:** 10.1371/journal.pone.0078625

**Published:** 2013-11-12

**Authors:** Sa Xiao, Guangyan Ni, Ragan M. Callaway

**Affiliations:** 1 Key Laboratory of Cell Activities and Stress Adaptations (Ministry of Education), School of Life Science, Lanzhou University, Lanzhou, Gansu, People's Republic of China; 2 Key Laboratory of Vegetation Restoration and Management of Degraded Ecosystems, South China Botanical Garden, Chinese Academy of Sciences, Guangzhou, Guangdong, People's Republic of China; 3 Division of Biological Sciences and the Institute on Ecosystems, The University of Montana, Missoula, Montana, United States of America; University of Alberta, Canada

## Abstract

Mono-dominance by invasive species provides opportunities to explore determinants of plant distributions and abundance; however, linking mechanistic results from small scale experiments to patterns in nature is difficult. We used experimentally derived competitive effects of an invader in North America, *Acroptilon repens*, on species with which it co-occurs in its native range of Uzbekistan and on species with which it occurs in its non-native ranges in North America, in individual-based models. We found that competitive effects yielded relative abundances of *Acroptilon* and other species in models that were qualitatively similar to those observed in the field in the two ranges. In its non-native range, *Acroptilon* can occur in nearly pure monocultures at local scales, whereas such nearly pure stands of *Acroptilon* appear to be much less common in its native range. Experimentally derived competitive effects of *Acroptilon* on other species predicted *Acroptilon* to be 4–9 times more proportionally abundant than natives in the North American models, but proportionally equal to or less than the abundance of natives in the Eurasian models. Our results suggest a novel way to integrate complex combinations of interactions simultaneously, and that biogeographical differences in the competitive effects of an invader correspond well with biogeographical differences in abundance and impact.

## Introduction

Competition can have strong effects on the distribution and abundance of plant species [1]–[3]. Our understanding of these competitive effects originates in part from field experiments along gradients of plant distributions [4]–[6] and productivity [7], [8], simultaneous comparisons of different mechanisms by which plants interact [9], and correlations between interaction strengths and natural abundances [10], [11]. Furthermore, exotic invasions provide unusual opportunities to explore the importance of competition as a determinant of plant distributions and abundance. This is because some exotic invaders become far more abundant and dominant in their non-native ranges and demonstrate exceptionally strong competitive effects against native species in the non-native range [12]–[14]. In a few cases invaders have been shown to elicit stronger competitive effects on species from the non-native range of the invader than species from the native range [15]–[17]. Also, Callaway et al. [18] compared the effects of neighbors on the growth and reproduction of *Centaurea stoebe* in Europe where it is native and uncommon to those in Montana where it is invasive and extremely abundant, and found strong negative competitive effects of neighboring vegetation on *C. stoebe* growth and reproduction in Europe. In contrast, identical experiments in Montana resulted in insignificant impacts of native competitors on *C. stoebe*.

In the same biogeographical context, studies of *Acroptilon repens* (hereafter *Acroptilon*) provide a different sort of opportunity to link competitive interactions to biogeographic differences in abundance. *Acroptilon* is native to Turkey, central Asia, and China where it can be a problematic weed in agricultural settings [19]. *Acroptilon* has been introduced throughout much of western North America and has been declared noxious in 16 western states (http://plants.usda.gov). *Acroptilon* appears to be highly competitive in its non-native range; nearly pure monocultures of this invader are not uncommon at local scales [20], and strong competitive and allelopathic effects of the species on North American natives have been reported [21]. Such nearly pure stands of *Acroptilon* rarely occur in at least two parts of its native range, Uzbekistan and Turkey (U. Schaffner & J. Littlefield, *unpublished data*), with dense stands restricted to regularly plowed orchards and highly disturbed roadsides, suggesting that *Acroptilon* may have lower impacts on its neighbors at home. In a comparison of three sites in each range, Callaway et al. [22] found that the biomass of *Acroptilon* in stands in North America was almost twice that in Uzbekistan where it is native. But more importantly, this difference in abundance translated to far greater differences between regions in the apparent impacts of *Acroptilon* on native species; the biomass of native species in *Acroptilon* stands was 25–30 times lower in the non-native range than in the native range. These biogeographic differences in abundance correspond with greenhouse experiments that have found *Acroptilon* to have stronger competitive and allelopathic effects on native North American species than on congeneric or confamilial native species from the native range of *Acroptilon* [23]. The mechanism for this is not known, but *Acroptilon* produces a polyacetelene [20], [24] which may allelopathically inhibit the growth of North American species more than European species.

Here we take a novel approach to predicting how small scale interactions among species such as described above might affect the long-term abundance and dynamics of species at the larger scale of community composition and diversity. Individual-based models provide a tool for predicting causal links between small scale interactions and larger scale ecological patterns [25]. Individual-based models provide a good opportunity to consolidate empirically measured complex interactions among multiple species and make predictions about how such interactions might correlate with the abundance of the same species in communities [26]–[29]. To our knowledge, individual-based models have been used only once with empirically derived indices of competitive interactions to construct these kinds of predictions [30]. Here we used experimentally derived competitive effects of the North American invader, *Acroptilon repens* from a previously published paper, Ni et al. [23], on a suite of species with which it co-occurs in its native range of Uzbekistan, and on a suite of species with which it occurs in its non-native ranges in North America, in individual-based models to predict the relative abundances of these species in each range. Specifically, we asked whether these competitive effects alone can predict very general patterns of *Acroptilon* dominance in its non-native range and the relative lack of dominance in its native range. Our hypothesis was that despite substantial variation in the competitive effects of *Acroptilon* on species from its native and non-native ranges these effects when modeled simultaneously would predict biogeographical difference in dominance.

## Materials and Methods

We used results reported by Ni et al. [23] to calculate Relative Interaction Intensities (RII [31] for the competitive effect [32] of *Acroptilon repens* on eight North American native species and nine species native to Uzbekistan. RII is calculated by dividing the difference between the biomass of the treatment and control by the sum of the biomass of the treatment and control ((B_T_ – B_C_)/(B_T_ + B_C_)). RII is a measure of the strength of interaction between species centered on zero with negative interactions (competition) indicated by values between 0 and −1, and positive interactions (facilitation) indicated by values between 0 and +1. RII allows for simple comparisons of interaction strength across taxa and treatments. Competition between species has two important components, competitive “effect” and competitive “response” or tolerance. Competitive responses of invaders to natives have been shown to be an important component of interactions among invaders and natives [32], but only measurements of RIIs for the effects of *Acroptilon* on other species were available. Thus experimentally derived competitive effects of *Acroptilon* were used as parameters in three models in which the competitive responses of *Acroptilon* to other species were held constant *within* a single model, but responses were varied *among* models in order to examine potential outcomes at different levels of competition responses from natives.

The methods of the experiment are reported in detail in Ni et al. [23]. In brief, they collected about 100 *Acroptilon* rhizomes near Yakima, Washington, USA and used these to grow single *Acroptilon* plants in 2.4 L pots (18 cm diameter and 22 cm depth) in a greenhouse at The University of Montana. Each pot was filled with 1.8 L pure sand (20/30 grit silica) at the bottom of the pots, and 0.5 L mixed sand and autoclaved soil (1∶1) at the top of the pots. *Acroptilon* plants were grown in the center of each pot, and 161 days after planting the rhizomes seeds of North American and Uzbek species were planted. Each was planted in each of three treatments: (1) grown alone as a control, (2) grown with one individual of the most closely related species from the other continent, and (3) grown with *Acroptilon* and the closely related species from the other continent in the same pot on either side of the *Acroptilon* plant which was in the center. Final replication in the analysis of competition between related North American and Eurasian species was n = 6 for *C. intybus* and the paired *Melilotus officinalis-Hedysarum boreale*, and n = 9–10 for the other 7 pairs. There were 295 pots in total. Once germinated, all species and treatments were grown in a naturally lighted greenhouse supplemented by 1,000 W Metal Halide lights from 7∶00–10∶00 and 16∶00–19∶00 h from December to March. Pots containing pairs of species were randomly placed on greenhouse benches and rotated among the benches once per week. For the first 2 weeks, all plants were watered every day until water drained from the pots, and afterwards pots were watered every other day until the end of the experiment. All plants were fertilized with 250 ml Miracle-Gro at 0.34 g/L every 4 weeks. Plants were harvested over a 2 week period after 11–13 weeks of growth, but all treatments for each pair of species was harvested within 1 day. After harvesting, the plants were dried at 60°C for 4 days and then weighed.

We used RIIs calculated from Ni et al. 's experiment [23] in an individual-based spatially-explicit dual-lattice model [29], [33], [34] with *Acroptilon* and native species occupying two overlapping two-dimensional lattices of similar sizes (100×100 cells). Each individual of *Acroptilon* and each individual of a native species occupied one cell in its own lattice. When reproduction occurred, an individual produced propagules that were identical to the parents. The total number of these propagules equalled the reproductive rate *r_A_* of the *Acroptilon* and the reproductive rate *r_N_* of the native species. We assumed *r_N_* was the same for all native species. Both types of propagules were dispersed sequentially to one of the patches that were randomly selected within its own lattice. The propagules were allowed to establish only in empty cells and the one arriving first occupied the cell. Thus all native species competed for space within the same lattice through lottery competition, i.e. not species-specific, among their propagules for the empty cells. We used a “wraparound” (torus) approach to avoid edge effects [35].

We assumed that the competitive effects of native species decreased *Acroptilon* survival rate linearly with the increase of the RII value of the native species on *Acroptilon*. Therefore, the survival rate of *Acroptilon* was:

 when it overlaps with native species *i*

 when it overlaps with empty celland where *S_Amax_* was the maximum survival rate of *Acroptilon*.

The native species lattice was composed of 8 species for North America and 9 species for Eurasian respectively (Table S1). We assumed the competitive effects of *Acroptilon* on native species also decreased their survival rates linearly with the increase of *RII* value of *Acroptilon* on native species. Therefore, the survival rate of population native species *i* is:

 when it overlaps with *Acroptilon*

 when it overlaps with empty celland where *S_Nmax_* was the maximum survival rate of native species. We assumed *S_Nmax_* was the same for all native species populations.

We used asynchronous updating in the model that worked in the following way. First a single individual of *Acroptilon* or native species was selected at random. Second, we determined whether the individual survived at a certain survival rate (with a survival probability *S_A_* and *S_Ni_* for the *Acroptilon* and native species, respectively). If the individual survived it reproduced and dispersed propagules. Each time step was made up of *N_A_* + *N_N_* of such updates, where *N_A_* and *N_N_* refer to the number of all individuals of *Acroptilon* and all individuals of the native species, respectively.

Since the initial population sizes of invaders are likely to be relatively small at the beginning of invasions, thus all simulations were started with only 100 individuals of *Acroptilon*. Initial conditions started with saturated communities of native species with each having the same number of individuals. All individuals of *Acroptilon* and native species were randomly dispersed across their own lattices.

All simulations were run for 10000 time steps in order to allow the system to reach a steady state. All measurements were determined as the mean values of 100 independent replicate runs for each time step. Parameters used in simulations were: *r*_A_  = 1, *r_N_*  = 1, *S_Amax_*  = 0.8, *S_Nmax_*  = 0.8, the RII values for the competitive effects of *Acroptilon* on native species are shown in Table S1, and we assume the competitive effects of native species on *Acroptilon* (RII*_N on__A_*) is same for all native species. The robustness of the model was tested with different combinations of parameters and results were qualitatively the same as for the combinations chosen here (data not shown). Simulations were performed in NetLogo [36], a powerful multi-agent modeling language particularly well suited for modeling complex systems that develop over time.

We statistically compared the mean extinction times for North American and Eurasian species with one way ANOVAs for each RII scenario using region as a fixed factor and the natural log of extinction times as the dependent variable (SPSS 19.0. 2010).

## Results

**Figure 1:**
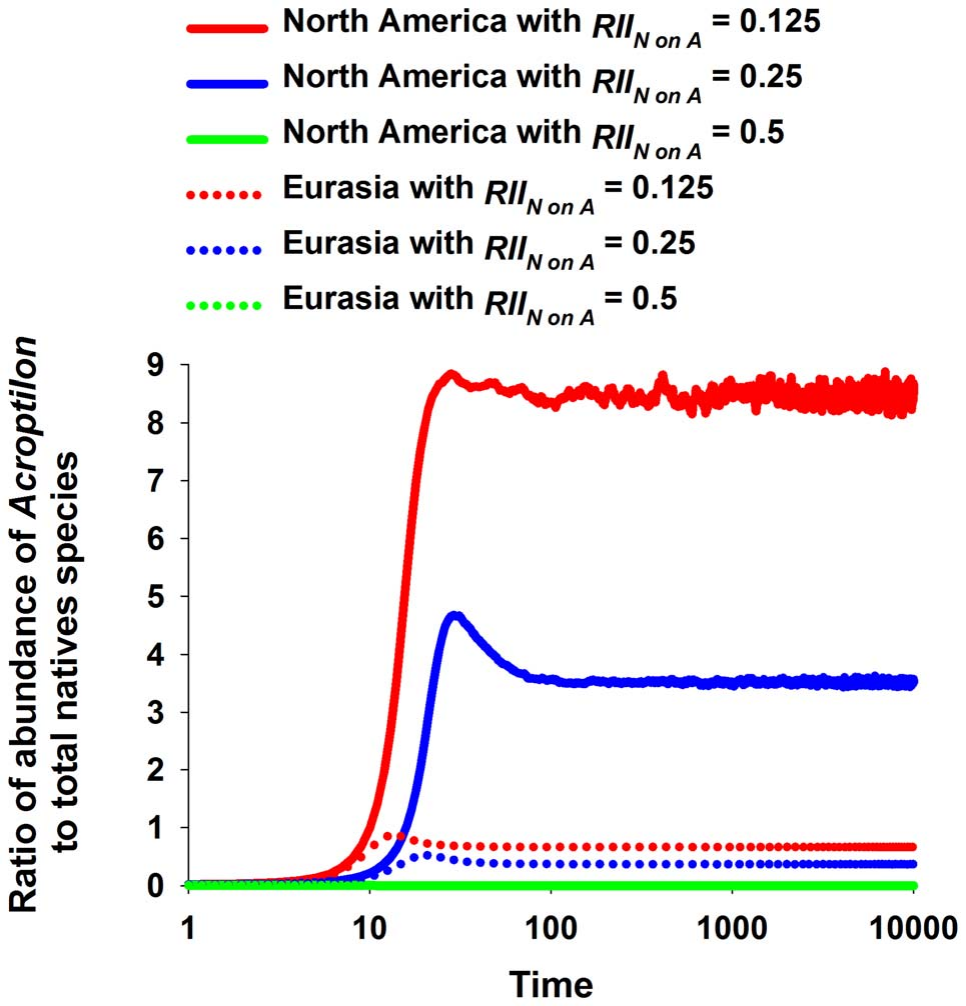
Ratios of abundances of *Acroptilon* to total native species in different competitive scenarios. In all scenarios the competitive effects of *Acroptilon* were derived from experiments reported in Ni et al. (2010). For scenarios for the native and non-native ranges the mean competitive effects of native species on *Acroptilon* were varied from RII = 0.125 to RII = 0.500. Scenarios for the North American range are represented by solid lines and Eurasian scenarios are represented by dashed lines. Note the log scale for the x-axis.

When the competitive effects of native species from *both* ranges on *Acroptilon* were low (RII*_N on__A_*  = 0.125), the empirically derived competitive effects of *Acroptilon* on other species predicted *Acroptilon* to be 9 times more proportionally abundant than natives in the North American scenario but proportionally equal to the abundance of natives in the Eurasian scenario (Fig. 1). This result supported our original hypothesis. When the same experimentally derived RIIs for *Acroptilon's* competitive effects were used, but with the competitive effects of all other species increased to RII*_N on__A_*  = 0.250, *Acroptilon* was approximately 4 times more abundant than natives in the North American scenario when the model reached equilibrium, but in the Uzbek scenario natives were approximately 2 times more abundant than *Acroptilon*. When RII*_N on__A_*  = 0.50 natives competitively excluded *Acroptilon* in both biogeographic scenarios. Thus, in the context of our original hypothesis, if the competitive *response* of *Acroptilon* is modified to reflect very strong competition from natives, then our original hypothesis was not supported.

**Figure 2:**
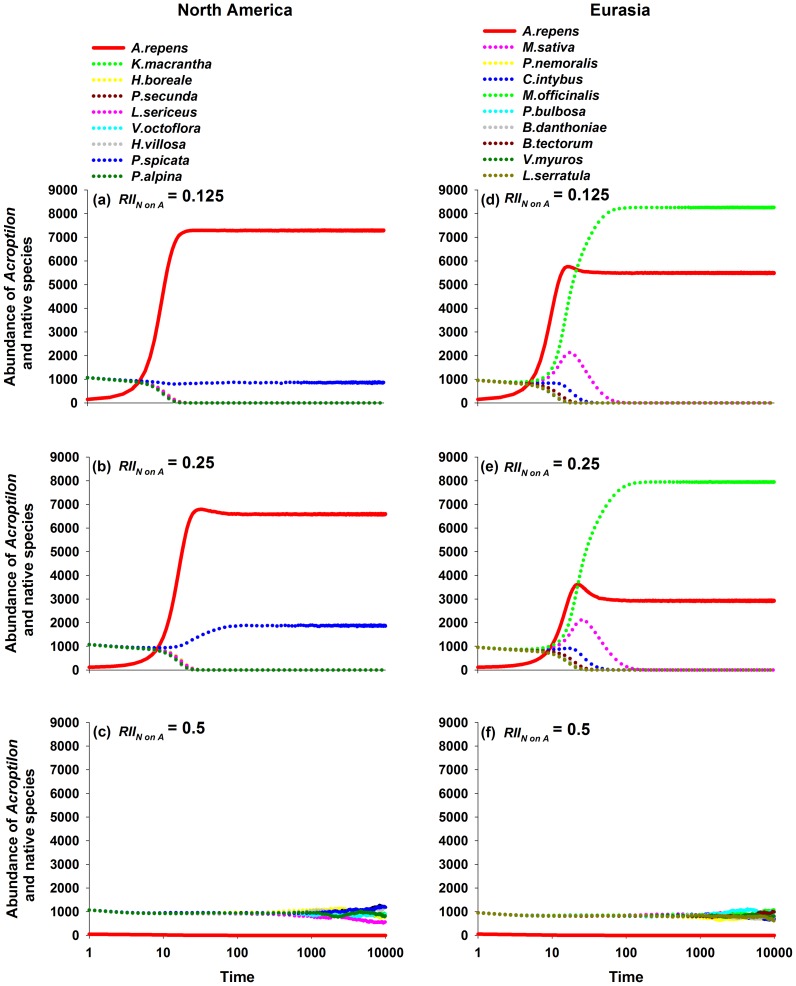
Abundances of *Acroptilon repens* and native species through time under different competitive scenarios. Abundances of *Acroptilon repens* and native species through time under different competitive scenarios for the native and non-native range of the invader. *Acroptilon* is represented by a solid line and native species are represented by dotted lines. Note the log scale on the x-axis.

These ratios *at equilibrium* were skewed by the survival of the most competitive native species in each region, due to the mathematical nature of the model, and thus only demonstrate patterns of relative and not directly quantitative interest. For example, when RII*_N on__A_*  = 0.125, all native species but *P. spicata* in North America and *M. officinalis* in Eurasia were ultimately eliminated from modeled communities (Fig. 2), and these two species was the least affected by *Acroptilon* in their respective regional groups in the experiments by Ni et al. [23]; i.e. the minimum value of RII*_A on__Ni_*. This pattern at equilibrium was because all native species compete with each other for space in the same lattice through lottery competition [37]–[39] among their propagules for the empty cells, so each empty cell is colonized in proportion to the abundance of each surviving species. Since species under the lowest competitive pressure from *Acroptilon* will inevitably realize the highest survival rate, which in turn will result in the largest reproductive rate of propagules for this species, thus bestowing this species with the greatest competitive ability for space. This inherent aspect of the model can create an unrealistic final outcome with this dominant competitor for space excluding all other species and become the single remaining species in scenarios.

Because the model produces unrealistic patterns of co-dominance at equilibrium, the responses of species at earlier points in the time sequences are important to consider. In the North American scenarios with RII*_N on__A_*  = 0.125 and 0.250, native richness started to decline at time steps 18 and 24 respectively, and they declined to one species at time steps 45 and 57. In the Eurasian scenarios with RII*_N on__A_*  = 0.125 and 0.250, native richness started to decline later, at time steps 21 and 33 respectively, and they declined to one species at time steps 118 and 187. At RII*_N on__A_*  = 0.125 the richness of Eurasian species was maintained at three species from time step 31 to 47, and at RII*_N on__A_*  = 0.250 from time step 52 to 77, approximately seven times longer than in the North American scenario. Eurasian diversity remained at two species from time step 48 to 117 at RII*_N on__A_*  = 0.125 and from 78 to 186 at RII*_N on__A_*  = 0.250, which was 26 to 33 times longer than in the North American scenario.

**Figure 3:**
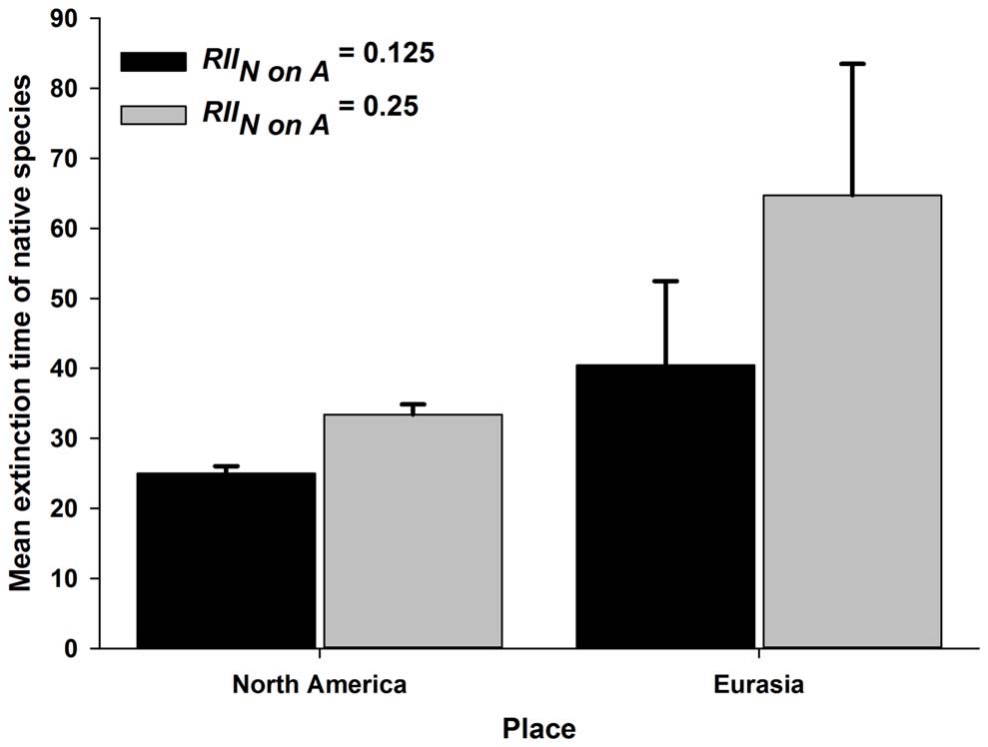
Mean extinction time for native species in different competitive scenarios. Mean extinction time for native species in different competitive scenarios (*RII_N on__A_*  = 0.15 and 0.25) between *Acroptilon* and species native to North America and Europe. *Pseudoroegneria spicata* in North America and *Melilotus officinalis* were not included because they were never eliminated from the models.

Taking another perspective, the mean number of model cycles survived by American species when RII*_N on__A_*  = 0.125 tended to be lower than that of Eurasian species (Fig. 3; ANOVA, F_region_ = 1.794; df = 1,14; P = 0.201), and when RII*_N on__A_*  = 0.250 the mean number of cycles survived by North American species was 48% lower than that of Eurasian species (Fig. 3; ANOVA, F_region_ = 4.833; df = 1,14; P = 0.047).

## Discussion

Using only experimentally derived differences in the competitive effects of an exotic invasive plant species on natives from the native and non-native ranges (derived from [23]), our individual-based models yielded relative abundances of *Acroptilon* and other species that were qualitatively similar to those observed in the field in the two ranges (see [22]). In its non-native range, *Acroptilon* can occur in nearly pure monocultures at local scales [20], whereas such nearly pure stands of *Acroptilon* appear to be much less common in its native range (U. Schaffner & J. Littlefield *unpublished data*).

Our model only incorporated empirically derived competitive *responses* of other species to *Acroptilon*, and not empirically derived measurements of the *effects* of native Eurasian species and North American species on *Acroptilon*. The competitive effects of exotic invasives on native species are likely to play an important role in successful invasion; however, there is evidence that the competitive responses of natives may be more important than their effects for preventing community collapse and shifts towards invasive monocultures. MacDougall and Turkington [32] found that the competitive response of an exotic invasive to natives determined long-term patterns of relative abundance in natural conditions of low fertility and limited disturbance, but argued that the role of competitive response and effect depended on resource availability and disturbance history. Incorporating and comparing empirically measured responses of invaders to competition from native species is likely to yield even more realistic links between competitive interactions and species abundances.

The RIIs used for these experiments are of course limited by the specific conditions in which the original experiments were conducted. Ni et al. [23] used simple pair-wise competition interactions in pots in controlled conditions, and these results can be very conditional [40]. Therefore, the outcomes we measured are likely to be different under different conditions. For example, *B. tectorum*, one of the Eurasian species, is a much better at high soil nitrogen availability [41], and there is ample evidence that other invasions are enhanced when resources are high [42]. Ni et al. [23] also autoclaved soil to avoid confounding soil feedback influences; however, intact soil biota could provide substantially different results. Competition can also vary with genotypic variation within a species, and the wide range of genotypic variation that is likely for some of our test species could create different competitive outcomes. Ni et al. [23] only used only *Acroptilon* from North America, and if the competitive effects of this species have evolved in the native range, the competitive effects of these plants would be weaker in other experiments. However, the species used in Ni et al. 's experiment were collected in communities in the field in both regions with the only criteria attempt to find species from the same genus or family. Another potential problem with the original experiment is that there were more annuals as competitors from Eurasia than from North America, and annuals may be unusually good short-term competitors in pots. In fact, annual Eurasian species were among the best pair-wise competitors. However, Ni et al. also tested the competitive interactions between all Eurasian-North American pairs and there was no regional difference overall. Also, since North American species and Eurasian species were competing against the same *Acroptilon* individuals, differences in the effects of *Acroptilon* were not based on biomass. Biomass-based effects are clearly important drivers of competitive outcomes [43], but our results suggest that they are not the only drivers.

The greater abundance of *P. spicata* as other species declined in modeled North American communities (compare Figure 2c to 2d), and the greater abundance of *M. officinalis* in Eurasian communities as other native were excluded, suggests a hypothesis in which indirect facilitation by *Acroptilon* occurs for some native species through the suppression of other native species. Among competitors, indirect facilitation can occur when a third (or more) species attenuates the intensity of competitive interactions between two others [44]–[46]. In our modeled scenarios, different native species varied in their competitive responses to *Acroptilon,* and all native species competed with each other for empty patches equally, creating a situation where all natives had the potential to be indirectly facilitated by the suppression of other native species by *Acroptilon*. The species with lowest competitive responses to *Acroptilon* were facilitated the most, and these were *P. spicata* in North America and *M. officinalis* in Eurasia.

Understanding general biogeographic differences in plant-herbivore interactions [47], plant-microbe interactions [48] or plant-plant interactions [49] has shed light on potentially important, but subtle evolutionary trajectories in the way species interact in communities [49]. Our integration of empirically measured competitive interactions contributes to understanding biogeographic patterns in ecology and evolution by quantitatively supporting the idea that the competitive effects of invasive plant species can be a major factor in determining their relative low abundance in native ranges and dominance in non-native ranges.

## Supporting Information

Table S1 **RII values of competitive effects of** ***Acroptilon repens*** **on 8 native species in North America and 9 native species in Eurasia respectively.**
(DOC)
