# Supplementary material for: Models of Experimentally Derived Competitive Effects Predict Biogeographical Differences in the Abundance of Invasive and Native Plant Species
Source: PLoS One. 2013 Nov 12;8(11):e78625. doi: 10.1371/journal.pone.0078625 (PMC3827048; doi:10.1371/journal.pone.0078625)
Supplement: Table S1 — RII values of competitive effects of Acroptilon repens on 8 native species in North America and 9 native species in Eurasia respectively. (DOC) [file pone.0078625.s001.doc]

Table S1. RII values of competitive effects of *Acroptilon repens* on 8 native species in North America and 9 native species in Eurasia respectively.

| North America | | Eurasia | |
| --- | --- | --- | --- |
| *Koeleria macrantha* | 0.60 | *Medicago sativa* | 0.01 |
| *Hedysarum boreale* | 0.60 | *Poa nemoralis* | 0.70 |
| *Poa secunda* | 0.66 | *Cichorium intybus* | 0.17 |
| *Lupinus sericeus* | 0.62 | *Melilotus officinalis* | -0.08 |
| *Vulpia octoflora* | 0.83 | *Poa bulbosa* | 0.60 |
| *Heterotheca villosa* | 0.80 | *Bromus danthonia* | 0.51 |
| *Pseudoroegneria spicata* | 0.37 | *Bromus tectorum* | 0.40 |
| *Poa alpina* | 0.75 | *Vulpia myuros* | 0.68 |
|  |  | *Lactuca serratula* | 0.64 |
